# Progress towards antibiotic use targets in eight high-income countries

**DOI:** 10.2471/BLT.20.270934

**Published:** 2021-04-29

**Authors:** Bryony Simmons, Koya Ariyoshi, Norio Ohmagari, Celine Pulcini, Benedikt Huttner, Sumanth Gandra, Giovanni Satta, Lorenzo Moja, Michael Sharland, Nicola Magrini, Marisa Miraldo, Graham Cooke

**Affiliations:** a Department of Infectious Disease, Imperial College London, London W2 1NY, England.; b Institute of Tropical Medicine, Nagasaki University, Nagasaki, Japan.; c Disease Control and Prevention Center, National Center for Global Health and Medicine, Tokyo, Japan.; d APEMAC, University of Lorraine, Nancy, France.; e Health Products Policy and Standards, World Health Organization, Geneva, Switzerland.; f Washington University School of Medicine, Washington University in St. Louis, St. Louis, United States of America.; g Institute of Infection and Immunity, St George’s University, London, England.; h Department of Economics and Public Policy & Centre for Health Economics and Policy Innovation, Imperial College Business School, London, England.

## Abstract

**Objective:**

To compare antibiotic sales in eight high-income countries using the 2019 World Health Organization (WHO) Access, Watch and Reserve (AWaRe) classification and the target of 60% consumption of Access category antibiotics.

**Methods:**

We analysed data from a commercial database of sales of systemic antibiotics in France, Germany, Italy, Japan, Spain, Switzerland, United Kingdom of Great Britain and Northern Ireland, and United States of America over the years 2013–2018. We classified antibiotics according to the 2019 AWaRe categories: Access, Watch, Reserve and Not Recommended. We measured antibiotic sales per capita in standard units (SU) per capita and calculated Access group sales as a percentage of total antibiotic sales.

**Findings:**

In 2018, per capita antibiotic sales ranged from 7.4 SU (Switzerland) to 20.0 SU (France); median sales of Access group antibiotics were 10.9 SU per capita (range: 3.5–15.0). Per capita sales declined moderately over 2013–2018. The median percentage of Access group antibiotics was 68% (range: 22–77 %); the Access group proportion increased in most countries between 2013 and 2018. Five countries exceeded the 60% target; two countries narrowly missed it (> 55% in Germany and Italy). Sales of Access antibiotics in Japan were low (22%), driven by relatively high sales of oral cephalosporins and macrolides.

**Conclusion:**

We have identified changes to prescribing that could allow countries to achieve the WHO target. The 60% Access group target provides a framework to inform national antibiotic policies and could be complemented by absolute measures and more ambitious values in specific settings.

## Résumé

**Objectif:**

Comparer les ventes d'antibiotiques dans huit pays à hauts revenus à l'aide de la classification AWaRe (Access, Watch et Reserve) mise en place en 2019 par l'Organisation mondiale de la Santé, ainsi que de l'objectif de consommation de 60% fixé pour les antibiotiques de la catégorie Access.

**Méthodes:**

Nous avons analysé les informations issues d'une base de données commerciales concernant la vente d'antibiotiques systémiques entre 2013 et 2018 en Allemagne, en Espagne, aux États-Unis d'Amérique, en France, en Italie, au Japon, au Royaume-Uni de Grande-Bretagne et d'Irlande du Nord et en Suisse. Nous avons réparti ces antibiotiques selon les catégories AWaRe 2019: Access (antibiotiques dont l'accessibilité est essentielle), Watch (antibiotiques à utiliser sélectivement) et Reserve (antibiotiques de réserve, non recommandés). Nous avons mesuré les ventes d'antibiotiques en unités standard (SU) par habitant, et calculé celles de la catégorie Access sous forme de pourcentage des ventes totales.

**Résultats:**

En 2018, les ventes d'antibiotiques par habitant étaient comprises entre 7,4 SU (Suisse) et 20,0 SU (France); la valeur médiane des ventes d'antibiotiques Access s'élevait à 10,9 SU par habitant (écart: 3,5-15,0). Les ventes par habitant ont diminué modérément au fil des ans entre 2013 et 2018. Le pourcentage médian des antibiotiques Access était de 68% (écart: 22–77%); dans la plupart des pays, leur pourcentage a augmenté entre 2013 et 2018. Cinq pays dépassaient l'objectif de 60%; deux autres s'en approchaient (> 55% en Allemagne et en Italie). La vente d'antibiotiques Access au Japon était faible (22%) en raison d'une forte consommation de macrolides et de céphalosporines orales.

**Conclusion:**

Nous avons constaté une évolution dans les prescriptions, ce qui pourrait permettre aux pays d'atteindre l'objectif de l'OMS. Une limite de consommation d'antibiotiques Access fixée à 60% pourrait servir d'orientation aux politiques nationales en la matière, et être complétée par des mesures absolues et des chiffres plus ambitieux dans des contextes spécifiques.

## Resumen

**Objetivo:**

Comparar las ventas de antibióticos en ocho países con ingresos altos utilizando la clasificación de acceso, vigilancia y reserva (AWaRe) de la Organización Mundial de la Salud (OMS) de 2019 y el objetivo de consumo del 60% de los antibióticos de la categoría de acceso.

**Métodos:**

Analizamos los datos de una base de datos comercial de ventas de antibióticos sistémicos en Francia, Italia, Japón, Alemania, España, Suiza, Reino Unido de Gran Bretaña e Irlanda del Norte y Estados Unidos de América durante los años 2013-2018. Clasificamos los antibióticos según las categorías AWaRe de 2019: acceso, vigilancia, reserva y no recomendado. Medimos las ventas de antibióticos per cápita en unidades estándar (SU) per cápita y calculamos las ventas del grupo de acceso como porcentaje de las ventas totales de antibióticos.

**Resultados:**

En 2018, las ventas per cápita de antibióticos oscilaron entre 7,4 SU (Suiza) y 20,0 SU (Francia); la media de las ventas de antibióticos del grupo de acceso fue de 10,9 SU per cápita (rango: 3,5-15,0). Las ventas per cápita disminuyeron moderadamente durante 2013-2018. La media del porcentaje de antibióticos del grupo de acceso fue del 68% (rango: 22-77%); la proporción del grupo de acceso aumentó en la mayoría de los países entre 2013 y 2018. Cinco países superaron el objetivo del 60%; dos países lo incumplieron por un escaso margen (> 55% en Alemania e Italia). Las ventas de antibióticos de acceso en Japón fueron bajas (22%), impulsadas por una venta relativamente alta de cefalosporinas orales y macrólidos.

**Conclusión:**

Hemos identificado cambios en la prescripción que podrían permitir a los países alcanzar el objetivo de la OMS. El objetivo del 60% del grupo de acceso proporciona un marco para informar las políticas nacionales de antibióticos y podría complementarse con medidas absolutas y valores más ambiciosos en entornos específicos.

## ملخص

**الغرض:**

مقارنة مبيعات المضادات الحيوية في ثماني دول مرتفعة الدخل باستخدام تصنيف منظمة الصحة العالمية (WHO) للإتاحة والمراقبة والاحتياطي (AWaRe) لعام 2019، والهدف المتمثل في استهلاك 60% من المضادات الحيوية من فئة الوصول.

**الطريقة:**

قمنا بتحليل البيانات من قاعدة البيانات التجارية لمبيعات المضادات الحيوية المنتظمة في فرنسا وإيطاليا واليابان وألمانيا وأسبانيا وسويسرا والمملكة المتحدة لبريطانيا العظمى وأيرلندا الشمالية والولايات المتحدة الأمريكية، خلال الأعوام 2013 إلى 2018. وقمنا بتصنيف المضادات الحيوية وفقًا لفئات 2019 AWaRe: الإتاحة، والمراقبة، والاحتياطي، وغير الموصى بها. قمنا بقياس مبيعات المضادات الحيوية للفرد في الوحدات القياسية (SU) للفرد، وكذلك حساب مبيعات مجموعة الإتاحة كنسبة مئوية من إجمالي مبيعات المضادات الحيوية.

**النتائج:**

في عام 2018، تراوحت مبيعات المضادات الحيوية للفرد من 7.4 وحدة قياسية (سويسرا) إلى 20.0 وحدة قياسية (فرنسا)؛ كان متوسط مبيعات المضادات الحيوية لمجموعة الإتاحة 10.9 وحدة قياسية للفرد (النطاق: 3.5 إلى 15.0). انخفض مبيعات الفرد بشكل معتدل خلال الفترة من 2013 إلى 2018. كانت متوسط النسبة المئوية للمضادات الحيوية لمجموعة الوصول 68% (النطاق: 22 إلى 77%)، وزادت نسبة مجموعة الوصول في معظم الدول بين عامي 2013 و2018. وتجاوزت خمس دول الهدف المتمثل في 60%؛ بينما أخفقت دولتان في تحقيق هذا الهدف بفارق ضئيل (> 55% في ألمانيا وإيطاليا). كانت مبيعات المضادات الحيوية المتاحة في اليابان منخفضة (22%)، نتيجةً للمبيعات المرتفعة نسبيًا من مركبات السيفالوسبورين والماكروليد الفموية.

**الاستنتاج:**

قمنا بتحديد تغييرات في الوصفات يمكنها أن تسمح للدول بتحقيق هدف منظمة الصحة العالمية إن هدف المجموعة المتاحة البالغ 60%، يوفر إطارًا لتوعية السياسات الوطنية للمضادات الحيوية، ويمكن استكماله بتدابير مطلقة، وقيم أكثر طموحًا في أوضاع محددة.، وقيم أكثر طموحًا في أوضاع محددة.

## 摘要

**目的:**

旨在根据 2019 年世界卫生组织 (WHO) 可用、慎用和备用 (AWaRe) 分级原则和 60% 可用级抗生素消费目标，比较 8 个高收入国家的抗生素销售情况。

**方法:**

我们分析了 2013 年至 2018 年期间德国、法国、美国、日本、瑞士、西班牙、意大利和英国全身性抗生素销售相关商业数据库的数据。我们根据 2019 年 AWaRe 分级原则对抗生素进行了分级：可用级、慎用级、备用级和不推荐使用。我们基于人均标准单位 (SU) 统计了人均抗生素销售额，并计算了可用级抗生素销售额占抗生素总销售额的百分比。

**结果:**

2018 年，人均抗生素销售额为 7.4 SU（瑞士）至 20.0 SU（法国）不等；可用级抗生素的平均销售额为人均 10.9 SU（范围：3.5–15.0）。2013-2018 年间人均销售额有所下降。可用级抗生素的中位百分比为 68%（范围：22–77%）；2013 年至 2018 年间，大多数国家的可用级抗生素消费比例有所上升。有 5 个国家超过了 60% 的消费目标；两个国家非常接近该目标值（德国和意大利均大于 55%）。鉴于其口服头孢菌素和大环内酯类药物销售额相对较高，日本的可用级抗生素的销售额较低 (22%)。

**结论:**

我们已确定处方更改方案，以帮助各国实现世卫组织的目标。60% 可用级抗生素消费目标为国家制定抗生素政策提供了框架，同时可针对特定环境设定一些绝对控制措施和宣扬更加着眼于未来的价值观。

## Резюме

**Цель:**

Сравнить продажи антибиотиков в восьми странах с высоким уровнем дохода, используя классификацию Всемирной организации здравоохранения (ВОЗ) 2019 года для антибиотиков AWaRe («доступ», «наблюдение» и «резерв»), а также прогресс в достижении цели увеличить долю глобального потребления антибиотиков на 60% в группе «доступ».

**Методы:**

Авторы проанализировали данные из коммерческой базы данных о продажах системных антибиотиков в Германии, Испании, Италии, Соединенном Королевстве Великобритании и Северной Ирландии, Соединенных Штатах Америки, Франции, Швейцарии и в Японии за 2013–2018 годы. Были классифицированы антибиотики по категориям AWaRe 2019: «доступ», «наблюдение», «резерв» и «нерекомендуемые». Авторы измерили продажи антибиотиков на душу населения в стандартных единицах (СЕ) на душу населения и рассчитали продажи в группе «доступ» как процент от общих продаж антибиотиков.

**Результаты:**

В 2018 году продажи антибиотиков на душу населения составляли от 7,4 СЕ (Швейцария) до 20,0 СЕ (Франция); медиана продаж антибиотиков в группе «доступ» составила 10,9 СЕ на душу населения (диапазон: 3,5–15,0). Продажи на душу населения в 2013–2018 годах умеренно снизились. Медиана процентной доли антибиотиков в группе «доступ» составила 68% (диапазон: 22–77%); процентная доля в группе «доступ» увеличилась в большинстве стран в период с 2013 по 2018 год. Пять стран превысили показатель в 60%; две страны чуть не упустили этот показатель (> 55% в Германии и Италии). Продажи антибиотиков в группе «доступ» в Японии были низкими (22%), что было обусловлено относительно высокими продажами пероральных цефалоспоринов и макролидов.

**Вывод:**

Мы определили изменения в назначениях, что позволит странам достичь целевого показателя ВОЗ. Достижение 60% в группе «доступ» служит основой для информационного обеспечения национальной политики в отношении антибиотиков, которую можно было бы дополнить абсолютными показателями и более амбициозными значениями в определенных условиях.

## Introduction

Antimicrobial resistance is a major threat to global health, endangering the ability to prevent and manage many common infectious diseases.^1^^,^^2^ High rates of use and misuse of antibiotics have contributed to selection pressures on drug-resistant strains of common pathogens, leading to a shift towards more expensive and broad-spectrum antibiotics.^3^ In 2015, the World Health Assembly adopted a Global Action Plan on Antimicrobial Resistance, calling for optimization of the use of antimicrobials.^4^ Key to optimization is to promote access to appropriate antibiotics while avoiding excess use.

The Access, Watch and Reserve (AWaRe) categorization is a tool introduced by the World Health Organization (WHO) to encourage antibiotic stewardship and to combat antimicrobial resistance.^5^ The categorization was first introduced in the 2017 WHO essential medicines list, in which key antibiotics were classified into three categories – Access, Watch and Reserve – according to their therapeutic and resistance profile.^6^ Access group antibiotics are defined as priority treatments recommended as first- and second-choice options for common infections that should be available and affordable in all countries. The Watch group contains broad-spectrum antibiotics with a higher resistance potential that are recommended for a specific, limited number of indications. The Reserve group includes antibiotics for multidrug-resistant infections that should be treated as last-resort options in highly specific patients and settings. Recognizing the role of the AWaRe as a policy tool, the WHO essential medicines list expert committee updated the classification in 2019 to categorize additional antibiotics into the three groups and to add a new category: Not Recommended.^7^^,^^8^ To reduce the use of Watch and Reserve group antibiotics, the WHO Thirteenth General Programme of Work 2019–2023 has adopted the following target to be reached by 2023: at least 60% of national antibiotic consumption should be from the Access group.^9^^,^^10^ Adoption of this target at the national level should help to inform and galvanize action and can be used to monitor progress, allowing for comparison of antibiotic stewardship efforts.

Global antibiotic consumption and prescribing behaviours have been described in studies in different countries, with variations in study years, data sources, breadth of analysis and patient populations (for example, paediatrics, hospital setting or overall population).^11^^–^^15^ In relation to AWaRe, antibiotic consumption by children has been measured against the 2017 AWaRe classification, with several countries falling short of the target of 60% use of Access group antibiotics.^16^^,^^17^ Similar findings have been observed in general population studies, including the WHO report on surveillance of antibiotic consumption and a recent multi-country analysis, both using data up to 2015.^15^^,^^18^ We aimed to add to this body of work by using sales data to assess patterns of antibiotic sales according to the 2019 AWaRe categories in eight high-income countries over the years 2013–2018. The objectives were to inform policy discussions and to assess progress towards the WHO Access group target.

## Methods

### Study design

To obtain indication of antibiotic consumption patterns, we used aggregate sales data as proxy. We analysed wholesale antibiotic sales data for 2013–2018 from eight countries to determine patterns of sales with reference to the 2019 WHO AWaRe classification.^8^^,^^19^ The countries included were: France, Germany, Italy, Japan, Spain, Switzerland, United Kingdom of Great Britain and Northern Ireland, and United States of America (USA). We chose the countries and years of observation based on the availability of data. The included countries are representative of high-income countries with large pharmaceutical markets, in regions with varying antibiotic resistance profiles and health-care contexts.

### Data sources

We used the IQVIA multinational integrated data analysis system database (IQVIA Inc., Durham, USA) to identify antibiotic sales. This commercial database tracks pharmaceutical sales by using national sales audits of manufacturers and wholesalers, through retail and non-retail channels.^20^^,^^21^ IQVIA standardizes the data to ensure they are nationally representative and to allow for comparability across markets. Table 1 shows the data sources and coverage of the database for our sample. We extracted national quarterly sales data of all single and combination antimicrobial medicines. For our data extract IQVIA aggregated the data across hospital and community sectors and captured sales by generic and nongeneric manufacturers; the data do not distinguish between indication or patient characteristics. IQVIA data are routinely used to understand sales volumes of pharmaceuticals and to conduct international comparisons.^11^^,^^15^^,^^17^^,^^22^ More details on the data set are provided in the author’s data repository.^23^

**Table 1:** National data sources and coverage of pharmaceutical sales in the IQVIA multinational integrated data analysis system database

Country and audit type	Data source	Market coverage
**France**		
Retail, sell-out	Pharmacies with computerized systems	100%
Hospital, consumption	Hospital trusts	
**Germany**		
Retail, sell-in	Pharmacies and wholesalers	100%
Hospital, consumption	Hospitals	
**Italy**		
Retail, sell-in	Pharmacies and wholesalers	99%
Hospital, consumption	Hospitals and local health authorities	
Direct to patient, consumption	Local health authorities	
**Japan**		
Retail, sell-in	Wholesalers	100%
Hospital, sell-in	Wholesalers	
**Spain**		
Retail, sell-in	Pharmacies and wholesalers	99%
Retail, sell-out	Pharmacies	
Hospital, consumption	Hospitals	
**Switzerland**		
Retail, sell-in	Deliveries of manufacturers, importers, and wholesalers	100%
Hospital, sell-in	Deliveries of manufacturers, importers, and wholesalers	
**United Kingdom**		
Retail, sell-out	Prescription data from pharmacies and direct sales panel	89%
Hospital, consumption	National Health Service beds	
**United States**		
Combined retail and hospital, sell-in	Wholesalers, mail service pharmacies, manufacturers, hospitals	100%

Notes: IQVIA use a combination of local audits at different points of the distribution chain to collect data on pharmaceutical volumes; collection methods vary by country subject to local conditions and data availability. Audit type shows the data collection methods for each country (that is, retail or hospital sector and the specific sales channel). Sell-in refers to the indirect sales from manufacturers or wholesalers to either retail pharmacies (retail, sell-in) or hospital pharmacies (hospital, sell-in). Sales or consumption to the consumer are measured as sales from retail pharmacies to the patient (retail, sell-out) or distribution from hospital pharmacies to the patient (hospital, consumption). Through the IQVIA multinational integrated data analysis system local audits are standardized to allow cross-country comparability and analysis. Market coverage gives the percentage of the total national pharmaceutical market which the combined audits represent. For countries with < 100% coverage, IQVIA adjust according to a proprietary algorithm to estimate the probable total sales value based on the market share of the participating sources, such that the extract estimates 100% of consumption.

Sources: IQVIA multinational integrated data analysis system (IQVIA Inc., Durham, USA)^20^ and national audit data sources.

To extract data on systemic antibiotic formulations from the sales database, we developed a comprehensive list of antibiotics used in human medicine. We used three sources: (i) WHO list of critically important antimicrobials for human medicine, 2018 edition; (ii) WHO anatomical therapeutic chemical (ATC) code J01 (antibacterials for systemic use); and (iii) WHO AWaRe classification, as presented in the 2019 WHO essential medicines list and in the WHO AWaRe classification database.^8^^,^^19^^,^^24^ Included antibiotics were those defined as antibacterials for systemic use; we excluded antifungal and antiviral drugs, drugs solely for tuberculosis and topical formulations. We reviewed the full IQVIA database to identify any potentially missed or nonclassified systemic antibiotics; national data were reviewed by country experts.

### Data analysis 

We estimated sales volumes in standard units (SU). SU refers to the number of standard dose units sold, where a dose is defined by IQVIA as one tablet or capsule for solid forms, one ampoule or vial for injectable forms, and 5 mL for syrup forms. We aggregated data at the year level by country and product. We defined each antibiotic product as Access, Watch, Reserve or Not Recommended, according to the 2019 AWaRe categorization.^8^^,^^19^ As the classification did not include all antibiotics identified in the sales data, we created a fifth group – unclassified – containing all systemic antibiotics not listed. We determined antibiotic pharmacological classifications using a combination of WHO ATC third- and fourth-level groups. Products and related AWaRe and antibiotic classifications are listed in the data repository.^23^

We used several metrics to explore sales patterns (Table 2). First, we calculated total antibiotic sales and antibiotic sales per person in each country, overall and by AWaRe category. We estimated per capita sales by linking total sales to total annual population estimates from the World Bank.^25^ Second, we calculated the percentage sales of each AWaRe category. Percentages were calculated as the number of SUs of antibiotics in each group divided by the total number of antibiotic SUs sold. The percentage of Access group antibiotics sold was described relative to the 60% target. We assessed sales trends between 2013 and 2018 using simple linear regression by country and overall. Overall trends were estimated using the population-weighted aggregate mean sales across all eight countries (details in the data repository).^23^ Next, we examined sales of specific antibiotic pharmacological classes, presenting the data as proportions of total antibiotic sales and proportions of the specific AWaRe category sale. Finally, to explore country-specific prescribing habits, we identified all products contributing to at least 3% of country-specific consumption in 2018 and all products for which one country contributed more than 60% of total consumption across our sample.

**Table 2:** Outcome definitions used in the study of antibiotic sales in eight high-income countries

Outcome	Level of analysis	Definition
Total annual sales, SU	Overall	Total sales of systemic antibiotics per year in SU
Annual sales per capita, SU per capita	Overall, by AWaRe category and by pharmacological class	No. of SU sold per year divided by the country–year population estimate derived from the World Bank^25^
Relative sales, % of total sales	By AWaRe category and by pharmacological class	No. of SU sold in each group divided by the total no. of antibiotic SU sold in the given year (multiplied by 100)
Relative sales, % of AWaRe category sales	By pharmacological class	No. of SU sold of the specific antibiotic class and AWaRe category divided by the no. of AWaRe category SU sold in the given year (multiplied by 100)
≥ 3% sales indicator	By individual antibiotic	Products accounting for ≥ 3% of country-specific consumption in 2018. Calculated as the no. of SU sold of each antibiotic divided by the total no. of SU by country in 2018
Single country indicator, ≥ 60% of total sales	By individual antibiotic	Products accounting for ≥ 60% of per capita consumption. Calculated as the no. of per capita SU sold for a particular antibiotic in each country divided by the total per capita SU over all countries in 2018. Calculated for all products identified in the ≥ 3% consumption list

AWaRe: Access, Watch and Reserve classification; SU: standard units.

Note: The unit for all analyses was at the country–year level. We identified AWaRe antibiotic categories according to the World Health Organization 2019 classification.^8^ We categorized pharmacological class according to the WHO anatomical therapeutic chemical third and fourth levels (see the data repository for classifications).^23^

We compiled additional data to analyse between-country differences in antibiotic sales. Country-level sociodemographic indicators were obtained from the World Bank.^25^ We obtained information on each country’s implementation of national antimicrobial policies from national reports and the WHO library of national action plans.^26^ Lastly, to validate the results, we compared country–year findings against European consumption data from the European Surveillance of Antimicrobial Consumption Network^27^ (see the data repository).^23^ We analysed all data using Stata, version 14.2 (StataCorp, College Station, USA).

## Results

**Fig. 1:**
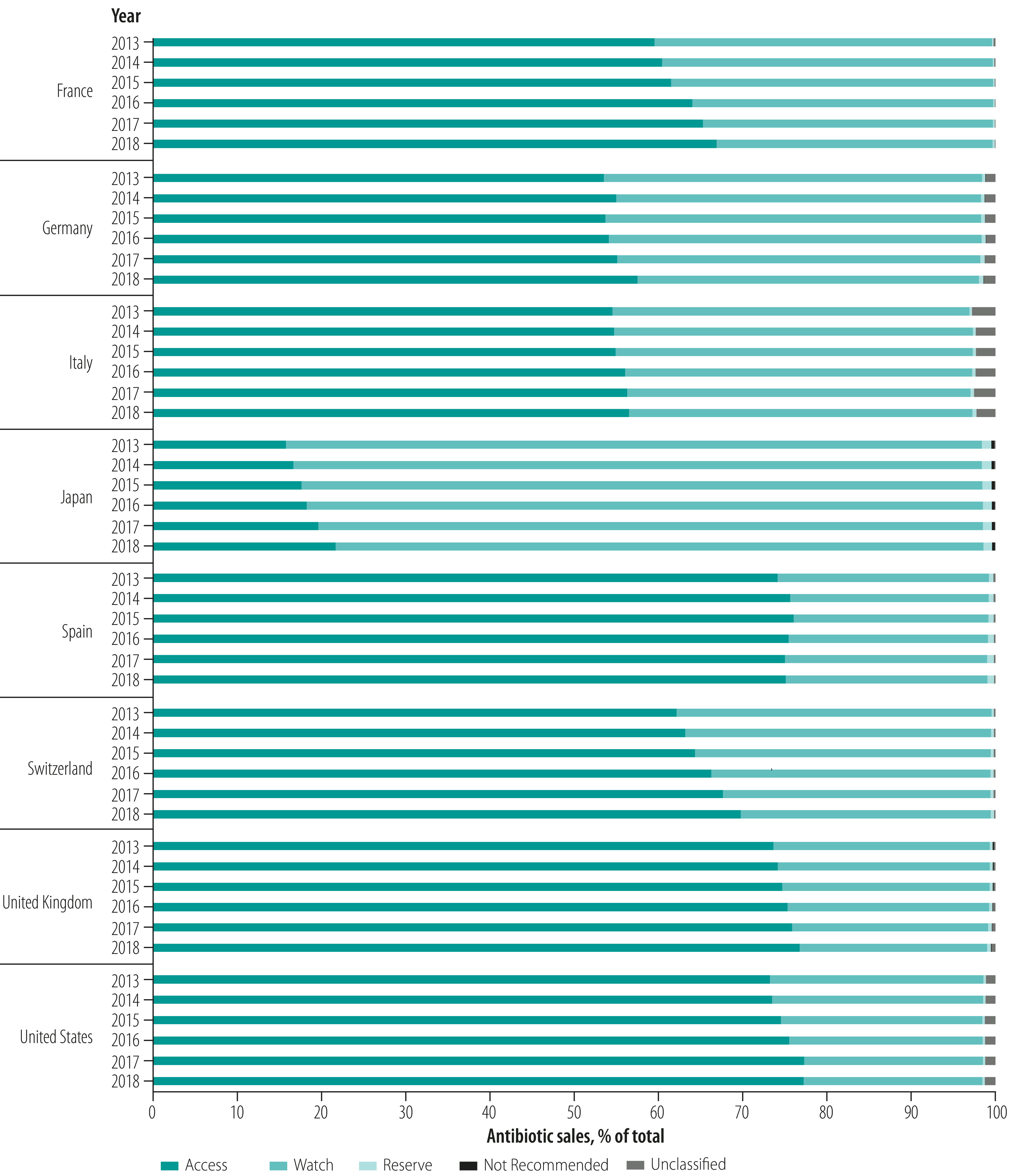
Proportion of total antibiotic sales in eight high-income countries by AWaRe category, 2013–2018

**Fig. 2:**
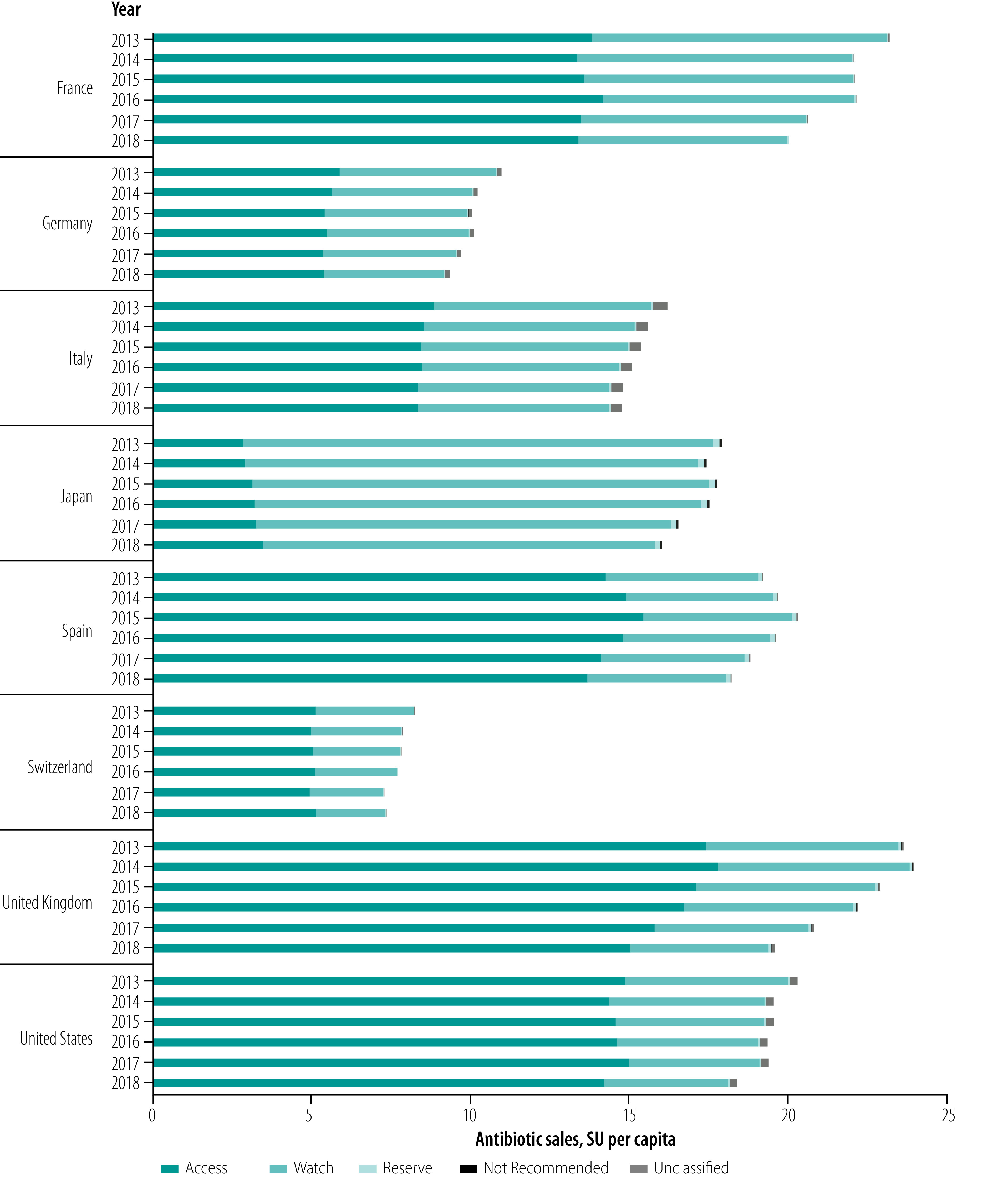
Per capita antibiotic sales in eight high-income countries by AWaRe category, 2013–2018

AWaRe: Access, Watch and Reserve classification; SU: standard units.

Note: The figure shows the number of standard units of antibiotics sold per capita, by 2019 World Health Organization AWaRe categories.^19^

**Fig. 3:**
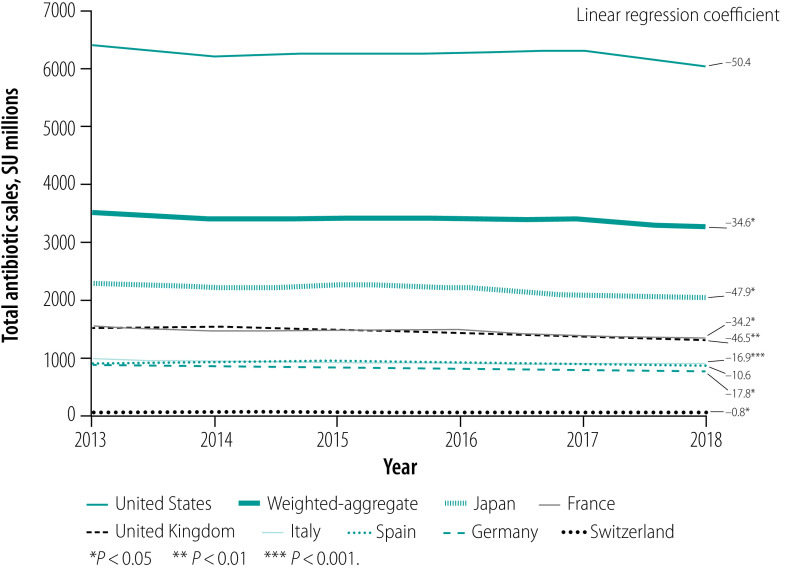
Trend in total annual antibiotic sales per capita in eight high-income countries, 2013–2018

**Fig. 4:**
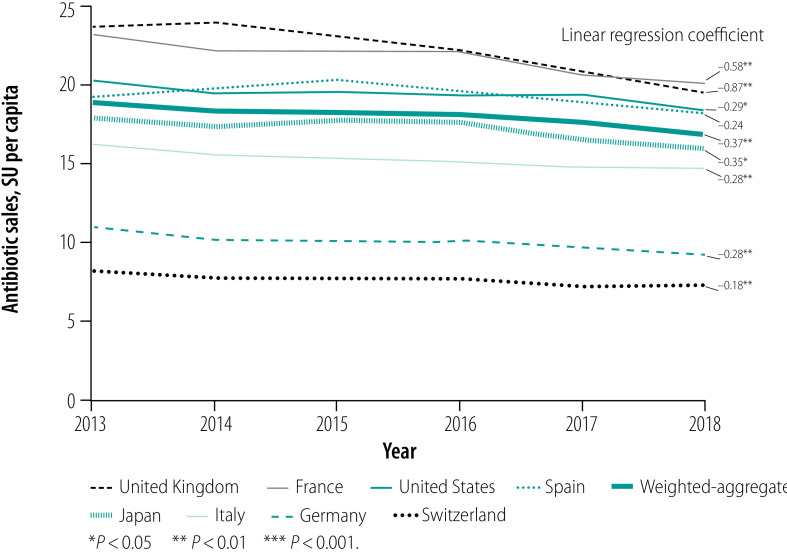
Trend in annual per capita antibiotic sales in eight high-income countries, 2013–2018

As of May 2020, all countries had implemented an antimicrobial national action plan and three countries had adopted AWaRe for antibiotic use surveillance (Germany, Switzerland and the United Kingdom; Table 3). France implemented a similar categorization in 2013, before AWaRe was published. In 2018, the median annual sales of systemic antibiotics were 1.1 billion SU, ranging from 0.1 billion in Switzerland to 6.0 billion in the USA. We found variability among the countries in 2018 levels of sales per capita, from the lowest in Germany and Switzerland (7.4 and 9.3 SU per capita, respectively) to the highest in France, Spain, the United Kingdom and USA (20.0, 18.2, 19.6 and 18.4 SU per capita, respectively). Overall antibiotic sales declined moderately between 2013 and 2018, both in terms of total sales and sales per capita (Fig. 1; Fig. 2; Fig. 3; Fig. 4). In 2018, the median sales of Access group antibiotics were 10.9 SU per capita (range: 3.5–15.0) and median sales of antibiotics in the Watch group were 4.4 SU per capita (range: 2.2–12.3).

**Table 3:** Country characteristics and antibiotic policies

Variable	France	Germany	Italy	Japan	Spain	Switzerland	United Kingdom	United States
**Sociodemographic characteristics**								
WHO Region	Europe	Europe	Europe	Western Pacific	Europe	Europe	Europe	Americas
Population, total in millions	67.0	82.9	60.4	126.5	46.7	8.5	66.5	327.2
GDP per capita, PPP current thousands of international dollars	45.9	54.3	42.1	43.3	40.9	68.9	46.2	62.6
Health expenditure per capita, PPP current thousands of international dollars	5.3	6.1	3.6	4.5	3.6	8.1	4.6	10.6
Health expenditure, % of GDP	11.2	11.2	8.8	10.9	8.9	12.2	9.8	16.9
**Antibiotic policies**								
National action plan on antibiotic use	Yes	Yes	Yes	Yes	Yes	Yes	Yes	Yes
Adopted AWaRe categorization in national policy	No, use similar categorization	Yes	No	No	No	Yes	Yes, adapted	No

AWaRe: Access, Watch and Reserve classification; GDP: gross domestic product; PPP: purchasing power parity; WHO: World Health Organization.

Note: Data shown are from the most recent year available between 2013 and 2018.

AWaRe: Access, Watch and Reserve classification.

Note: The figure shows the percentage of the total number of systemic antibiotic standard units sold, by 2019 World Health Organization AWaRe categories.^19^

SU: standard units.

Notes: Numeric estimates provided to the right of each trend line are the linear regression coefficient and can be interpreted as the estimated average annual change in the outcome for each country and for the weighted-aggregate. Weighted-aggregate is the population-weighted mean over the eight countries. Values are presented in the data repository.^23^

SU: standard units.

Notes: Numeric estimates provided to the right of each trend line are the linear regression coefficient and can be interpreted as the estimated average annual change in the outcome for each country and for the weighted-aggregate. Weighted-aggregate is the population-weighted mean over the eight countries. Values are presented in the data repository.^23^

In 2018, the median percentage of Access group antibiotics sold was 68.3%, varying from 21.6% (439/2030 million SU) of total sales in Japan to 76.8% in the United Kingdom (1000/1302 million SU) and 77.2% in the USA (4649/6020 million SU; Table 4; Fig. 1). When evaluated against the 60% target, five of the eight countries (France, Spain, Switzerland, the United Kingdom and USA) exceeded the 60% threshold and two were within 5.0 percentage points (Germany and Italy: 57.5% and 56.5%, respectively); only Japan’s figure was substantially lower. 

**Table 4:** Trends in consumption of Access group antibiotics in eight high-income countries, 2013–2018

Variable	No. of Access group antibiotics/total no. of antibiotics sold (%), million SU								Population-weighted mean %^a^
	France	Germany	Italy	Japan	Spain	Switzerland	United Kingdom	United States	
**Year**									
2013	912/1532 (59.5)	474/885 (53.5)	532/976 (54.5)	360/2286 (15.7)	665/897 (74.1)	41/67 (62.1)	1117/1517 (73.6)	4700/6418 (73.2)	59.0
2014	886/1466 (60.4)	455/828 (55.0)	518/948 (54.7)	369/2220 (16.6)	692/915 (75.6)	41/64 (63.2)	1150/1551 (74.2)	4575/6225 (73.5)	59.7
2015	905/1472 (61.5)	441/821 (53.7)	512/933 (54.9)	397/2261 (17.6)	718/944 (76.0)	42/65 (64.3)	1114/1492 (74.7)	4676/6274 (74.5)	60.4
2016	948/1482 (64.0)	450/831 (54.1)	513/915 (56.0)	405/2228 (18.2)	688/912 (75.5)	43/65 (66.3)	1098/1458 (75.3)	4724/6256 (75.5)	61.3
2017	900/1380 (65.3)	442/802 (55.1)	505/897 (56.3)	411/2099 (19.6)	658/877 (75.0)	42/62 (67.6)	1044/1376 (75.9)	4876/6307 (77.3)	62.6
2018	898/1343 (66.9)	445/774 (57.5)	504/892 (56.5)	439/2030 (21.6)	640/852 (75.1)	44/63 (69.7)	1000/1302 (76.8)	4649/6020 (77.2)	63.5
**Statistical analysis**									
Annual trend^b^	1.54	0.59	0.45	1.11	0.07	1.53	0.61	0.93	0.92
*P*-value for trend	< 0.001	NS	< 0.01	< 0.001	NS	< 0.001	< 0.001	< 0.01	< 0.001

NS: not significant; SU: standard units.

^a^ The population-weighted mean is based on the sales data for all eight countries.

^b^ The average annual trend shows the country-specific annual change between 2013 and 2018, derived from linear regression (separate models by country) and can be interpreted as the estimated average annual change in the outcome for each country and for the weighted-aggregate.

**Fig. 5:**
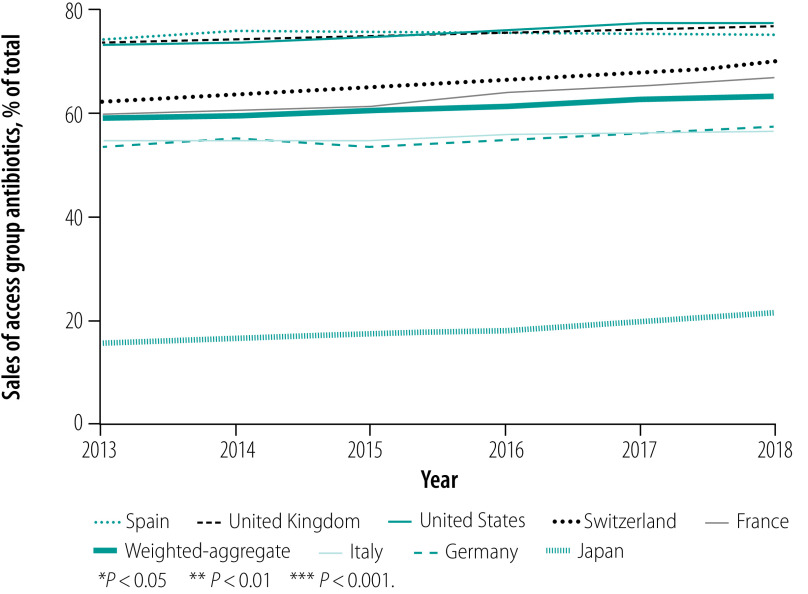
Trend in percentage of Access group antibiotics sold in eight high-income countries, 2013–2018

Notes: The linear regression coefficients for the trends are presented in Table 4. Weighted-aggregate is the population-weighted mean over the eight countries. Values are presented in the data repository.^23^

The percentage of Access group antibiotics sold increased over 2013–2018 in all countries by a population-weighted average annual change of 0.9 percentage points (*P* < 0.001; Table 4; Fig. 5). Variation arose mainly between the Access and Watch groups, with sales of these categories accounting from a median of 98.8% of sales (range: 97.3–99.7). Reserve group antibiotics made up less than 1.5% of sales in all countries and years (median in 2018: 0.4%; range: 0.2–1.2). Not Recommended and unclassified products accounted for a small proportion of total sales (median in 2018: 0.5%; range: 0.1–2.2).

Sales by pharmacological class and AWaRe category are shown in the data repository.^23^ Access group penicillins accounted for a median 70.7% (range: 47.6–86.6) of Access group antibiotics sold in 2018 and 48.8% (range: 13.9–64.2) of total antibiotic sales. The sales pattern of Access group penicillin varied between countries, with most classified as extended-spectrum penicillins (median: 31.8% of Access group; range: 19.3–52.0) or extended-spectrum with a β-lactamase inhibitor (median: 22.0% of Access group; range: 8.8–67.0). The United Kingdom had a higher use of narrow-spectrum β-lactamase-sensitive and -resistant penicillins at 27.4% (357/1302 million SU) of total sales. Japan had lower relative sales of Access group penicillins than other countries at 13.9% (283/2030 million SU) of total sales. Sulfonamides were the second most sold Access group antibiotic (median: 8.0% of Access group; range: 3.3–15.0; 4.0% of total; range: 2.2–7.9) followed by tetracyclines (median: 7.2% of Access group; range: 2.0–13.8; 5.2% of total; range: 1.1–10.7). The most frequently sold Watch group antibiotics were cephalosporins (median: 24.9% of Watch group; range: 2.0–43.2; 6.8% of total; range: 0.4–33.3), fluoroquinolones (median 24.6% of Watch group; range: 10.5–42.5; 8.9% of total; range: 2.3–12.6), and macrolides (median: 25.4% of Watch group; range: 17.2–42.2; 6.7% of total; range: 5.2–23.8). In Japan, Watch cephalosporins sold more than in other countries both in terms of percentage of Watch group antibiotics (43.2%; 675/1562 million SU) and overall antibiotic sales (33.3%; 675/2030 million SU), driven by high sales of third-generation cephalosporins. The most widely sold Reserve group antibiotics were polymyxins (median: 47.8% of Reserve group; range: 3.1–72.0) and oxazolidinones (median: 20.5% of Reserve group; range: 2.7–45.3); these made up a very small proportion of overall sales (< 0.5%).

Amoxicillin was the only product that accounted for ≥ 3% of sales in all countries assessed (median: 19.0% of total consumption; range: 10.0–34.0; 2.8 SU per capita); amoxicillin/clavulanic acid sales were ≥ 3% in all countries except Japan (median 14.4%; range: 1.9–37.7; 2.0 SU per capita) as shown in the data repository.^23^ In total, nine products were sold primarily in one country (defined as one country accounting for ≥ 60% of total consumption); three were sold exclusively in one country: pristinamycin (France), cefcapene pivoxil (Japan) and oxytetracycline (the United Kingdom).

For external validity, we compared comparable country–year results against European Centre for Disease Prevention and Control network data for France, Italy, Spain and the United Kingdom. These results showed a strong correlation and support the validity of our results (see the data repository).^23^

## Discussion

It is encouraging that in 2018 several of the countries studied achieved the WHO 60% Access group target, with a median percentage sales of Access group antibiotics across the eight countries of 68%. Of the three countries not meeting the target, Italy and Germany narrowly missed it, while Japan’s proportion was notably lower. Most countries made progress in optimizing antibiotic sales between 2013 and 2018, both in terms of an increase in the relative sales of Access group products and a decrease in per person sales of antibiotics.

The AWaRe classification and associated target provides a framework for simplified and standardized antibiotic surveillance and has the support of the G20 group of governments and central bank governors.^28^^,^^29^ Some of the studied countries have already adopted AWaRe for surveillance of antibiotics in an effort to translate international guidance into effective stewardship.^28^^,^^30^ To most effectively inform practice, England and Scotland in the United Kingdom have adapted the index to their local context, re-categorizing certain products, based on local resistance profiles, antibiotic use and the health-care setting.^30^ Preceding AWaRe, France initiated a similar categorization with associated targets and incentivized quality improvement mechanisms.^31^^,^^32^

In 2018, WHO released the first global report applying the 2017 AWaRe classification to evaluate 2015 levels of antibiotic use.^18^ We build upon this report and other studies, using the updated 2019 AWaRe categorization and more recent data through to 2018, and providing a detailed analysis of specific consumption patterns.^15^^,^^18^ In comparison with these studies, we observed marginally higher relative sales of Access group antibiotics, while the intra-country differences and time trends remained similar. Our analysis highlights cross-country differences in per capita antibiotic sales. For example, consistent with other reports,^11^ Germany and Switzerland sold relatively small quantities of antibiotics. Across countries, most heterogeneity was observed between Access and Watch group antibiotics; sales of Reserve category and Not Recommended products were low. Cross-country comparisons allow for some inferences about the appropriateness of antibiotic sales but should be interpreted with caution due to differences in burden, resistance profiles, treatment guidelines and health systems.^13^^,^^33^^,^^34^

Japan stands out as having a differing pattern of antibiotic consumption. We found lower relative sales of Access group broad-spectrum penicillins and higher sales of Watch category antibiotics, predominantly driven by high relative sales of third-generation cephalosporins and, to a lesser extent, macrolides and fluoroquinolones. These findings corroborate other studies of antimicrobial sales in Japan.^35^^,^^36^ The differences in sales patterns have several potential explanations. Many of the products sold in Japan were of Japanese origin and some products were rarely sold in other countries. This pattern might suggest a difference in regulatory requirements that discourage Japanese companies from seeking market authorization outside of Asia and likewise delaying or preventing uptake of products of non-Japanese origin. Japanese authorities may also prefer marketing strategies focusing on the domestic market.^37^^,^^38^ Differences in resistance patterns, patient demographics and cultural factors might also contribute to a different uptake of products in Japan. The Japanese antimicrobial national action plan promotes optimization of drug use and targets a reduced consumption of cephalosporins, fluoroquinolones and macrolides by 50% in 2020 from the 2013 baseline level.^39^ We observed progress towards this target with around 15% reduction of cephalosporin, fluoroquinolone and macrolide sales, but further action is required to meet targets.

The 60% Access group target provides a simple metric to monitor Access and promote responsible antibiotic use, but an emphasis on relative consumption alone could have unintended consequences on absolute consumption. In Germany, the Access group target was narrowly missed. The target could be achieved by a switch from second-generation Watch group cephalosporins to the Access group first-generation cephalosporins, but likewise it could be met through unnecessarily increasing sales of Access group products. Japan, despite a low Access group index, had relatively low total antibiotic sales and relatively low reported rates of antibiotic resistance. The European Centre for Disease Prevention and Control list of indicators for monitoring antibiotic consumption utilizes an absolute measure as the primary indicator of consumption to consider the amount of antimicrobials used.^40^ Secondary indicators, such as the ratio of broad-spectrum to narrow-spectrum antibiotics, overlap largely with the AWaRe Watch and Reserve group categories. Our findings and complementary assessment tools illustrate the importance for future stewardship policies in combining the Access group target with measures of total absolute consumption to give a more nuanced view of antibiotic stewardship.

Our study had some limitations. We estimated sales using SU, a standardized measure within the data source representing a single dose unit of sales. This method differs from the WHO consumption surveillance, which uses the ATC/defined daily dose method of calculation.^18^ SU provide an easily interpretable and standardized measure that does not require assumptions of sales which may not be correct in all settings and case-mixes of patients.^17^^,^^41^ However, the use of SU may limit comparisons across populations, particularly when dosing regimens and durations are variable, possibly biasing results towards the sales of antibiotics with longer durations or frequencies of dosing.^17^ As in previous studies, we observed a strong correlation between defined daily doses and SU when comparing our results with European Surveillance of Antimicrobial Consumption Network data for European countries. Our findings show similar trends to the WHO report^18^ and other recent studies.^11^^,^^14^^,^^17^

The use of sales data has certain limitations. Foremost, we did not study individual-level consumption. We could not determine patient characteristics and indications for treatment: factors critical to determining the appropriateness of prescribing and antibiotic use. Second, sales data may not be representative of the entire market – particularly for those countries where the data covers less than 100% of the pharmaceutical market – and the IQVIA algorithm to produce nationally representative estimates is not publicly available. Third, it may not be possible to disaggregate data by sector, facility and subnational geographies. Finally, the use of aggregate sales data provides a simple and standardized proxy for antibiotic consumption but should be complemented by analysis of data sources that enable conclusions to be drawn about the appropriateness of antibiotic use at the patient level.^18^ Future studies should assess consumption using sources such as prescribing data, dispensing records, and insurance and reimbursement records. Indicators using these data could more closely reflect the quality of antimicrobial prescribing.

Finally, while including a smaller subset of countries has allowed for a more in-depth analysis of antibiotic sales practices in this selection of highly developed countries, the findings need to be complemented by global data and more heterogeneous settings. Global increases in antibiotic consumption have been shown to be driven by rapid increases in consumption of Watch group antibiotics, particularly in low- and middle-income countries, highlighting the need for broader analyses to support stewardship efforts.^15^ In addition, future analyses could more deeply explore factors associated with differences in sales, how AWaRe is being adopted and adapted to national contexts and stewardship plans, and the impact of the introduction of AWaRe on antibiotic sales and resistance patterns.

Monitoring antibiotic consumption is an essential policy action highlighting potential areas where changes are needed to reduce the risk of antimicrobial resistance. As countries adopt the WHO AWaRe framework, there is a need to assess changes in antibiotic sales and use over time, and whether the WHO Access target is sufficient to preserve antibiotic efficacy across a range of infections or should be expanded. All countries should consider adapting the AWaRe classification and target to individual settings, with country-specific targets unambiguously reported in antimicrobial national action plans. Additional metrics, such as those focusing on absolute consumption, and more ambitious targets are needed to better adapt appropriateness of antibiotic use, particularly in mature health-care systems.
